# Rat TNF-Related Apoptosis-Inducing Ligand (rTRAIL)-Expressing Rat-Adipose-Derived Stem Cells (rADSC) Moderately Limit Growth of Rat Mammary Cancer Cells—A Pilot Study

**DOI:** 10.3390/cimb48090941

**Published:** 2026-09-15

**Authors:** Wiktor Pascal, Mateusz Gotowiec, Antoni Smoliński, Paweł K. Włodarski

**Affiliations:** 1 Department of Methodology, Medical University of Warsaw, 1b Banacha Street, 02-091 Warsaw, Poland; mateusz.gotowiec@wum.edu.pl (M.G.); s082979@student.wum.edu.pl (A.S.); 2 Doctoral School, Medical University of Warsaw, 81 Żwirki i Wigury Street, 02-091 Warsaw, Poland; 3 Department of Histology and Embryology, Medical University of Warsaw, 5 Chałubińskiego Street, 02-004 Warsaw, Poland; pawel.wlodarski@wum.edu.pl

**Keywords:** biologic brachytherapy, TRAIL, TNFSF10, adipose-derived stem cells, breast cancer, cell-based gene therapy, lentiviral transduction, apoptosis

## Abstract

Background: Adipose-derived stem cells (ADSCs) are readily harvestable, tumour-homing cells that have been engineered as vehicles for antitumour agents. TNF-related apoptosis-inducing ligand (TRAIL) selectively triggers apoptosis in many cancer cells through death receptors DR4/DR5, while sparing most healthy cells. To date, only human TRAIL has been studied in such delivery systems; a fully homologous rat model (rat TRAIL delivered by rat ADSC) has not been tested, despite being essential for future syngeneic in vivo studies. Methods: In this pilot, exploratory study, we lentivirally transduced rat ADSC (rADSC) to express myc-tagged rat TRAIL, characterized the expression of TRAIL-pathway components in rADSC and three rat mammary carcinoma cell lines (RBA, HH-16.cl.4, SHZ-88) at the mRNA and protein levels, and assessed the antitumour effect of rTRAIL-rADSC using recombinant TRAIL dose–response, supernatant transfer, direct co-culture, and nine-day insert co-culture assays. Results: rADSCs were successfully transduced and produced cell-associated rat TRAIL (66.2 pg per 50,000 cells versus 4.4–5.7 pg in controls; *p* < 0.05); this ELISA measures total cell-associated (intracellular plus membrane-anchored) TRAIL rather than soluble TRAIL. rADSC expressed markedly higher levels of the decoy molecule osteoprotegerin and of DR4/DR5 than the cancer cells. Two of three cancer lines (RBA, HH-16.cl.4) were susceptible to rat TRAIL, and rTRAIL-rADSC reduced their growth and increased the proportion of dead cells, with morphological features compatible with both apoptosis and necrosis, most markedly in HH-16.cl.4; the effect was separable from the empty-vector control in HH-16.cl.4 but not in RBA. Conclusions: In this pilot study, homologous rat TRAIL delivered by rADSC is feasible and increases cell death in TRAIL-susceptible rat mammary cancer cells, but the effect is moderate at most, contact-dependent, and in one of two susceptible lines not attributable to TRAIL itself. These findings define both the potential and the limitations of a fully translational rat model of TRAIL-based biologic brachytherapy and provide a foundation for future in vivo studies.

## 1. Introduction

Breast cancer is the most frequently diagnosed malignancy worldwide, with an estimated 2.26 million new cases in 2020 [1]. The risk of cancer recurrence after mastectomy differs from 7 to 13 percent within the five years depending on the stage [2]. To reduce this risk, adjuvant chemotherapy or radiotherapy is frequently administered after surgery; however, both modalities carry systemic and local complications. Therefore, there is a clear need for alternative, localized strategies that lower recurrence with fewer adverse effects.

Biologic brachytherapy is a concept first described by Michaels et al. [3] and recently summarized by our team [4] that uses molecular biology tools to make a limited volume of tissue produce a chosen therapeutic agent—typically a cytokine, protein, or oligonucleotide. By locally transfecting a tissue fragment, such as a surgical flap, the tissue is induced to secrete the desired protein directly at the target site, offering a route to deliver local therapy to the tumour bed. However, controlling genetic modification across an entire tissue bulk is difficult; transducing cells in vitro before delivery can overcome this limitation.

**Figure 1:**
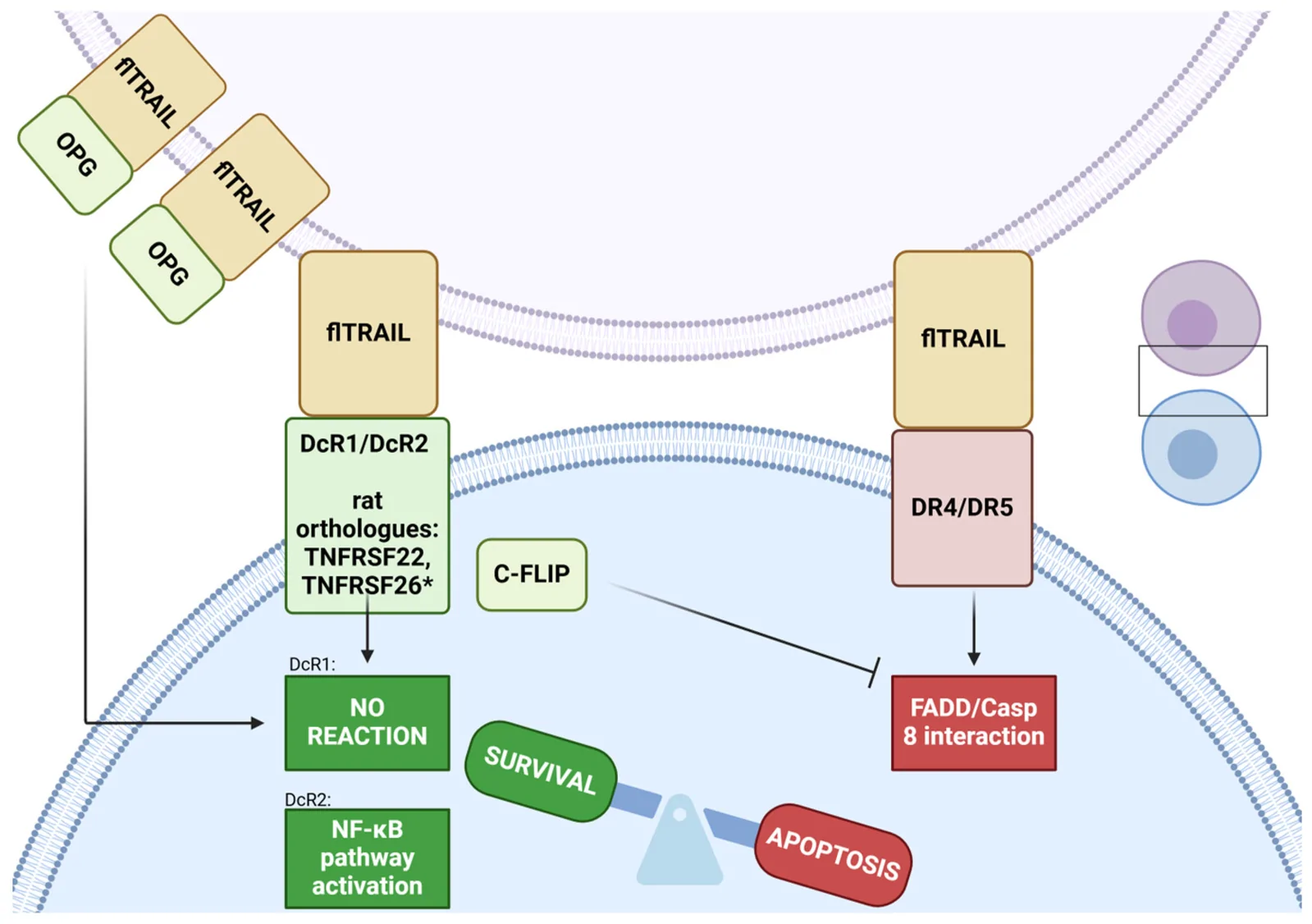
TNF-related apoptosis-inducing ligand (TRAIL) can trigger pro-survival or apoptotic signals depending on the receptor availability on the cell surface and its surroundings. * TNFRSF26 is an orphan receptor. Created in BioRender. Pascal, W. (2024) https://BioRender.com/o20h195.

Tumour necrosis factor (TNF)-related apoptosis-inducing ligand (TRAIL) is a homotrimeric protein that signals through the death receptors DR4 and DR5 to induce apoptosis preferentially in tumour cells while leaving most non-tumoral cells unharmed, underlining its potential as a therapeutic protein [5]. In healthy and TRAIL-resistant cancer cells, this pathway is blocked by transmembrane decoy receptors (DcR1, DcR2) and other molecules such as soluble osteoprotegerin (OPG) or through interference with caspase-8 by the FLICE-inhibitory protein (C-FLIP) following the activation by TRAIL/DR4/DR5 complexes [5]. When not disturbed, caspase 8 participates in the activation of caspase-3, further leading to cell apoptosis (Figure 1). It has been established that some breast cancer cell lines are sensitive to TRAIL, whereas others are relatively resistant; however, the mechanisms responsible for it are not fully understood [6]. Furthermore, the protein always used was human TRAIL and a rat analogue and its effectiveness were never tested before. Therefore, we aim to compare TRAIL pathway component expression between rat ADSC and rat mammary gland cancer cells. ADSC were chosen as the comparator group—rather than normal mammary epithelial cells—because they serve as the therapeutic vehicle and must themselves resist TRAIL-induced apoptosis while delivering it to the tumor. Identifying differences in death receptors (DR4/DR5), decoy receptors, and anti-apoptotic inhibitors between the vehicle (ADSC) and the target (cancer cells) is directly relevant to predicting therapy efficacy and explaining variable anticancer outcomes across cell lines.

Mesenchymal stem cells (MSC) are a promising tool in cancer therapy due to their tumour-homing capacity, immunomodulatory properties, and regenerative abilities; when engineered to express anticancer agents, they serve as effective local delivery vehicles [7,8]. In the past, bone marrow-derived mesenchymal stem cells were successfully transduced to produce TRAIL locally and kill cancer cells in vitro and in vivo with limited success [9,10]. However, due to its difficult collection process, a much more promising subdivision of MSC are adipose-derived stem cells (ADSCs). They are easily harvestable from patient’s fat deposits as a part of stromal–vascular fraction (SVF) cells and can help in regeneration after tumour resection [11]. What is more, ADSCs are widely applied for their potential to differentiate into osteocytes, adipocytes, neural cells, vascular endothelial cells, cardiomyocytes, pancreatic β-cells, and hepatocytes [12]. In the past, ADSCs have already been engineered to deliver TRAIL locally in primary brain tumour models, melanoma, hepatocellular carcinoma and cervical cancer with positive results in vitro and/or in vivo [13,14,15,16]; however, their usage in breast cancer therapy is limited because certain breast cancer cell lines display partial resistance to TRAIL-induced apoptosis [17]. This resistance is partial and cell-line-dependent (i.e., relative rather than absolute), and has been mechanistically linked to constitutive endocytosis of DR4 and DR5, which reduces surface receptor availability [16]. Notably, the lower total DR4/DR5 expression detected in mammary cancer cells compared to ADSC in our study is consistent with this endocytosis-driven reduction in steady-state receptor levels. ADSCs also have tumour-oriented homing capacity and thus wide potential as an efficient vehicle in patient-tailored cancer therapy. This tumour-directed migration and incorporation of ADSCs have been demonstrated in a number of pre-clinical studies using in vitro and in vivo animal tumour models [18,19]. On the other hand, it is postulated that ADSCs may favour breast cancer growth and recurrence via alternative pathways [20]. Recent studies have found that ADSCs promote tumoursphere formation in vitro and in vivo in breast cancer, increasing recurrence rate, rendering ADSC-mediated therapies not valid [21,22]. Therefore, characterizing differences in TRAIL pathway components between ADSCs and cancer cells is essential: ADSCs must express sufficient decoy receptors or anti-apoptotic molecules to survive TRAIL exposure, while susceptible cancer cells must express functional DR4/DR5 with limited decoy mechanisms. Such profiling directly predicts which cancer cell populations are likely to respond to TRAIL delivered by ADSCs, thereby explaining the variable anticancer activity observed across different tumor types and cell lines.

In this study, we aim to create a model of lentivirally transduced rat TRAIL-expressing adipose-derived stem cells and test its efficiency against rat mammary gland cancer cells.

## 2. Materials and Methods

### 2.1. Cell Culture

Rat adenocarcinomas of mammary gland cell lines (RBA, HH-16.cl.4, SHZ-88) were purchased from different suppliers (RBA: CRL-1747 ATCC, Manassas, VA, USA, HH-16.cl4: ACC 358, Leibniz-Institut DSMZ, Braunschweig, Germany, SHZ-88: 305209 CLS Cell Lines Service, Eppelheim, Germany) and cultured according to supplier’s instructions depending on the cell line. RBA cells were cultured in DMEM+ Glutamax medium (Gibco, Thermo Fisher Scientific, Waltham, MA, USA) and HH-16.cl.4 and SHZ-88 cells in RPMI 1640 medium (Gibco, Thermo Fisher Scientific, Waltham, MA, USA) with 10% FBS (Sigma-Aldrich, Burlington, MA, USA) and a penicillin–streptomycin mix (Gibco, Thermo Fisher Scientific, Waltham, MA, USA). Rat adipose-derived stem cells (ADSC, 10RA-001) derived from inguinal white fat pad were purchased (10RA-001, iXCells Biotechnologies, San Diego, CA, USA) and cultivated in DMEM/F12+Glutamax (Gibco, Thermo Fisher Scientific, Waltham, MA, USA) medium containing 10% FBS and penicillin–streptomycin mix. Human embryonic kidney cell line (Lenti-X 293T, cat no: 632180, Takara Bio, Clontech, Mountain View, CA, USA) was purchased and cultured in DMEM+Glutamax medium with 10% FBS and antibiotic mix. All cells were tested for presence of mycoplasma using LookOut^®^ Mycoplasma PCR Detection Kit (Sigma-Aldrich, Burlington, MA, USA) prior to experimental procedures and were found negative (Figure S2).

### 2.2. Plasmid Cloning, Lentiviral Transduction of rADSCs

Total RNA was isolated from female Sprague–Dawley rat kidney using RNeasy Mini Kit (Qiagen, Hilden, Germany) and its quality was assessed (A260/A280 > 1.8) using a spectrophotometer (Nanodrop 2000, Thermo Fisher Scientific, Waltham, MA, USA). A cDNA strand was synthesized using reverse transcriptase (GoScript, Promega, Woods Hollow, WI, USA). Full-length rat TRAIL gene with myc-tag at N-terminus and flanking sequences for reconstruction of restrictase sites was amplified from the prepared cDNA strand using high-fidelity polymerase (CloneAmp Hifi, Takara Bio, Clontech, Mountain View, CA, USA). The following primers were used: forward primer: 5′-TATTTCCGGTGAATTCATGGAGCAGAAACTCATCTCTGAAGAGGATCTGCCTTCCACCGGGAAC-3′; reverse primer: 5′-GAGAGGGGCGGGATCCCTAGTTAATTAAAAAGGCTCCAAAGAAACTGG-3′.

Plvx-CMV-IRES-Zsgreen1 plasmid, a gift from prof. Dominika Nowis from Laboratory of Experimental Medicine, Medical University of Warsaw, Warsaw, Poland containing Zsgreen1 fluorescent marker was linearised using BamH1 HF and EcoR1 restriction enzymes (BamH1: Fast Digest, Thermo Fisher Scientific, Waltham, MA, USA, EcoR1: New England Biolabs, Ipswich, MA, USA) and later used for cloning of transgenes (InFusion HD, Takara Bio, Clontech, Mountain View, CA, USA). Rat TRAIL was cloned into the multiple cloning site of the vector. Plasmids were collected using MidiPrep Kit (Qiagen, Hilden, Germany) according to manufacturer’s instructions and the cloning was first confirmed using backbone colony PCR and restriction excision using previously used restrictases and later using Sanger sequencing. Lentiviral particles were produced using a fourth-generation lentiviral packaging system (VSV-G envelope, Lenti-X, Takara Bio, Clontech, Mountain View, CA, USA). Lentiviral supernatants were concentrated tenfold using the Lenti-X Concentrator Kit (Takara Bio). Titre was approximated using a p24 capsid-antigen test (Lenti-X Go-Stix, Takara Bio, Clontech, Mountain View, CA, USA). rADSCs were transduced in antibiotic-free medium containing 8 μg/mL polybrene (Thermo Fisher Scientific, Waltham, MA, USA) with lentiviral particles carrying either the TRAIL gene or the empty plasmid (fluorescent protein only) for 24 h. To increase transduction efficiency, cells were centrifuged at 560× *g* for 1 h after addition of the lentivirus. After 24 h, the medium was replaced with a fresh medium. Transduction efficiency was assessed at 24, 48, and 72 h post-transduction, and long-term expression was monitored for up to 21 days using a fluorescence microscope (PALM Robo, Zeiss, Oberkochen, Germany).

### 2.3. RNA Isolation, Quantitative Polymerase Chain Reaction and Western Blotting

Total RNA was isolated from confluent cells using miRNeasy Kit (Qiagen) and its quality (A260/A280 > 1.8) was assessed using a microspectrophotometer (Nanodrop 2000, Thermofisher). cDNA was synthesized using reverse transcriptase (GoScript, Promega, Woods Hollow, WI, USA).

The following primers were used in this study: GAPDH_F: TGGGAAGCTGGTCATCAAC, GAPDH_R: GCATCACCCCATTTGATGTT, OPG_F: TGCAAAAGATGTCCGGATGG, OPG_R: GTTGCTTCTCTGTTTCCGGA, DR5_F: GGGTTTATCTCTCTTGGACGC, DR5_R: AGATCAACTTCAGGGCATCAG, CFLIP_F: GAAGCCCTCACCTTGTTTCC, CFLIP_R: CCAGTTCAATCACCAGGTCC, TNFRSF22_F: TTTTGCTGCCCATGTACCAC, TNFRSF22_R: ACATCAGACAAATGCCCAGC, TNFRSF26_F: GACTCCAAGCAAGGACTGTCA, TNFRSF26_R: GGTTGAGACGGTGTCGTTGA.

Furthermore, for protein analyses, confluent cells were lysed in RIPA buffer (50 mM Tris-HCl, 150 mM NaCl, 2 mM EDTA and 1% Triton X-100) containing protease and phosphatase inhibitors. Protein concentration was determined using the Pierce BCA Assay (Thermo Fisher Scientific, Waltham, MA, USA). Protein samples were separated on 10% sodium dodecyl sulfate-polyacrylamide (SDS-PAGE) gels under reducing conditions (+β-mercaptoethanol) and transferred to a PVDF membrane (Immobilon^®^-P, Merck, Darmstadt, Germany). Between 15 and 40 μg of protein was loaded per lane. The membrane was blocked for 1 h with 5% non-fat dry milk and then incubated with primary antibodies overnight at 6 °C. After three 10 min washes in TBST, the membrane was incubated with the secondary antibody in 5% non-fat dry milk for 1 h at room temperature and washed again. Reactive proteins were visualized with an ECL reagent (Thermo Fisher Scientific, Waltham, MA, USA) according to the manufacturer’s protocol. Relative protein expression was quantified using ImageJ 1.53v programme-based calculation of target band intensity compared to a beta-actin band.

Antibodies used: OPG mouse monoclonal NB100-56505 (Novus Biologicals, Centennial, CO, USA), rabbit DR5 ab16329 (Abcam, Cambridge, UK), rabbit DR4 ab8414 (Abcam, Cambridge, UK), mouse beta-actin sc-47778 (Santa Cruz Biotechnology, Dallas, TX, USA), mouse anti-myc, mouse anti-rabbit HRP conjugated sc-2357 (Santa Cruz Biotechnology, Dallas, TX, USA), donkey anti-mouse HRP conjugated sc-2314 (Santa Cruz Biotechnology, Dallas, TX, USA). Concentrations of the antibodies used: DR5—1/250, beta-actin—1/50, antimouse—1/1000, OPG—1/1000, DR4—1/500, antirabbit—1/1000, myc—1/500.

### 2.4. ELISA for Determination of TRAIL Concentration in Transduced rADSCs

Transduced (mycTRAIL-expressing and Zsgreen1-expressing) and non-transduced ADSCs were collected at 72 h post-transduction, frozen–thawed three times and then used to estimate rat TRAIL concentration using rat TRAIL/TNFSF10 Quantikine ELISA Kit (R&D Systems, Minneapolis, MI, USA) according to manufacturer’s instructions. The results were compared with a standard curve (TRAIL pg/mL) and expressed as pg per 50,000 cells. Because the cells were lysed by three freeze–thaw cycles before assay, this measurement reports total cell-associated TRAIL (intracellular plus membrane-anchored) and not soluble TRAIL released into the medium; soluble TRAIL was not quantified directly, and its functional availability was instead assessed indirectly by the conditioned-medium transfer assay described in Section 2.5.

### 2.5. Proliferation Assay Using Recombinant TRAIL and Supernatants from Transduced ADSCs

Cells were cultured in 96-well plates in 100 μL of medium, and rat recombinant TRAIL (Lifespan Biosciences, Lynnwood, WA, USA) at concentrations ranging from 0 to 100 ng/mL was added 24 h after seeding. In parallel, 100 μL of supernatant collected from cells 72 h after transduction (negative control, Zsgreen1-ADSC, or mycTRAIL-ADSC) was added to the cancer cells. Each treatment group was run in triplicate. Cells were incubated with TRAIL for 24 h, after which viability was assessed by a colorimetric assay (CellTiter 96 AQueous One Solution Cell Proliferation Assay, Promega, Woods Hollow, WI, USA), which contains the tetrazolium compound [3-(4,5-dimethylthiazol-2-yl)-5-(3-carboxymethoxyphenyl)-2-(4-sulfophenyl)-2H-tetrazolium] and the electron-coupling reagent phenazine ethosulfate (PES). Absorbance was measured at 490 nm.

### 2.6. Assessment of Live, Apoptotic and Necrotic Cells as Well as Apoptosis Induction Through Direct Co-Culture of Mammary Gland Cancer Cells and rADSCs and Supernatant from Transduced ADSCs

Rat mammary gland cancer cells were seeded in low numbers (10,000 cells per well) in 8-well cell culture slide (SPL Life Sciences, Pocheon-si, Republic of Korea) for fluorescent microscopy assessment or in high numbers (500,000 cells per well) in a 6-well plate (Corning, NY, USA). Twenty-four hours later, appropriate amounts of 72 h transduced rADSCs were seeded onto them or the supernatant from 72 h transduced rADSCs was added in a 1:1 proportion to the medium. After another 24 h, cells were either treated with Hoechst 33342 (Abcam, Cambridge, UK) according to manufacturer’s instruction and 1 μg/mL Propidium Iodide (Thermo Fisher Scientific, Waltham, MA, USA) or cells were lysed for Western blotting as previously described. For assessment of apoptotic and necrotic cells, pictures of three random fields (20×) were taken using a fluorescent microscope, Nikon ECLIPSE Ti-TimeLapse (Nishi-Ōi, Tokyo, Japan) using the following filters: DAPI filter (358 nm excitation/461 nm emission) for Hoechst 33342, Green Excitation filter (450–490 nm excitation/515 nm emission) for Zsgreen1 green fluorescent protein and PE/Texas Red channel for Propidium Iodide. The number of all cells was based on the umber of nuclei stained by Hoechst 33342; condensed blue stained nuclei were counted as apoptotic cells and cells with red staining were counted as necrotic cells, similarly to Pengnam et al. [22]. For Western blotting, the following antibodies were used: rabbit polyclonal Cleaved Caspase-3 (Asp175) 1/1000 (9661S, Cell Signalling Technology, Danvers, Massachusetts, USA), mouse monoclonal beta Actin Monoclonal Antibody (BA3R), HRP 1/1000 (MA5-15739-HRP, Thermo Fisher Scientific, Waltham, MA, USA) and mouse anti-rabbit HRP conjugated 1/1000 sc-2357 (Santa Cruz Biotechnology, Dallas, TX, USA).

### 2.7. Indirect Co-Culture of Mammary Gland Cancer Cells and rADSCs

Rat mammary gland cancer cells were seeded at a low density (200 cells per well) in the lower chamber of a 24-well plate (Transwell, Corning, Corning, NY, USA). Twenty-four hours later, 10,000 freshly transduced or control rADSCs were seeded into each 3 μm insert (Transwell, Corning, Corning, NY, USA). After a further 24 h (i.e., 96 h after rADSC transduction), the cells on the inserts were photographed using a fluorescence microscope. The two populations were co-cultured for nine days, after which the number of cells in the lower chamber was determined by 0.5% crystal violet (Warchem, Zakręt, Poland) staining.

### 2.8. Statistical Analysis

Statistical analyses and plots were performed and designed in GraphPad Prism 10.3.1 (GraphPad Prism, San Diego, CA, USA) with the threshold of statistical significance set at *p* ≤ 0.05. Throughout this study, an independent (biological) replicate denotes an experiment performed on a separate day using a separate rADSC transduction, from a distinct cell passage, with independently prepared cancer-cell platings; technical replicates denote parallel wells within a single such experiment. Unless stated otherwise, n refers to the number of independent biological replicates, and technical replicates were averaged within each biological replicate before statistical analysis. Individual biological data points are shown in all bar graphs, and any experiment performed as a single biological replicate is reported descriptively without inferential statistics. In the co-culture experiments, the comparison between mycTRAIL-rADSC and Zsgreen1-rADSC (empty-vector cells carrying an identical lentiviral transduction but no TRAIL) was pre-specified as the TRAIL-specific contrast, and the comparison between Zsgreen1-rADSC and untransduced rADSCs as the transduction-specific contrast. Depending on the data availability and number of groups, different statistical tests were used: (1) one-way ANOVA for comparison of more than two groups when a single factor was present or (2) two-way ANOVA with Dunnett’s multiple-comparisons test for comparison of more than two groups when more than a single factor was present.

## 3. Results

### 3.1. rADSCs Can Be Lentivirally Transduced to Produce Rat TRAIL

The results are presented as a sequential chain. We first establish the therapeutic tool and show that the TRAIL it produces is cell-associated (Section 3.1); we then profile TRAIL-pathway components to explain why the vehicle survives its own payload and to predict which cancer lines should be susceptible (Section 3.2); we test that prediction with recombinant rat TRAIL, which both selects the susceptible lines and identifies a resistant control line (Section 3.3); finding little activity in cell-free conditioned medium, consistent with a membrane-anchored ligand, we move to contact-based assays (Section 3.3 and Section 3.4); and finally we use nine-day co-culture with empty-vector controls to separate the TRAIL-attributable effect from that of the transduced vehicle (Section 3.5).

**Figure 2:**
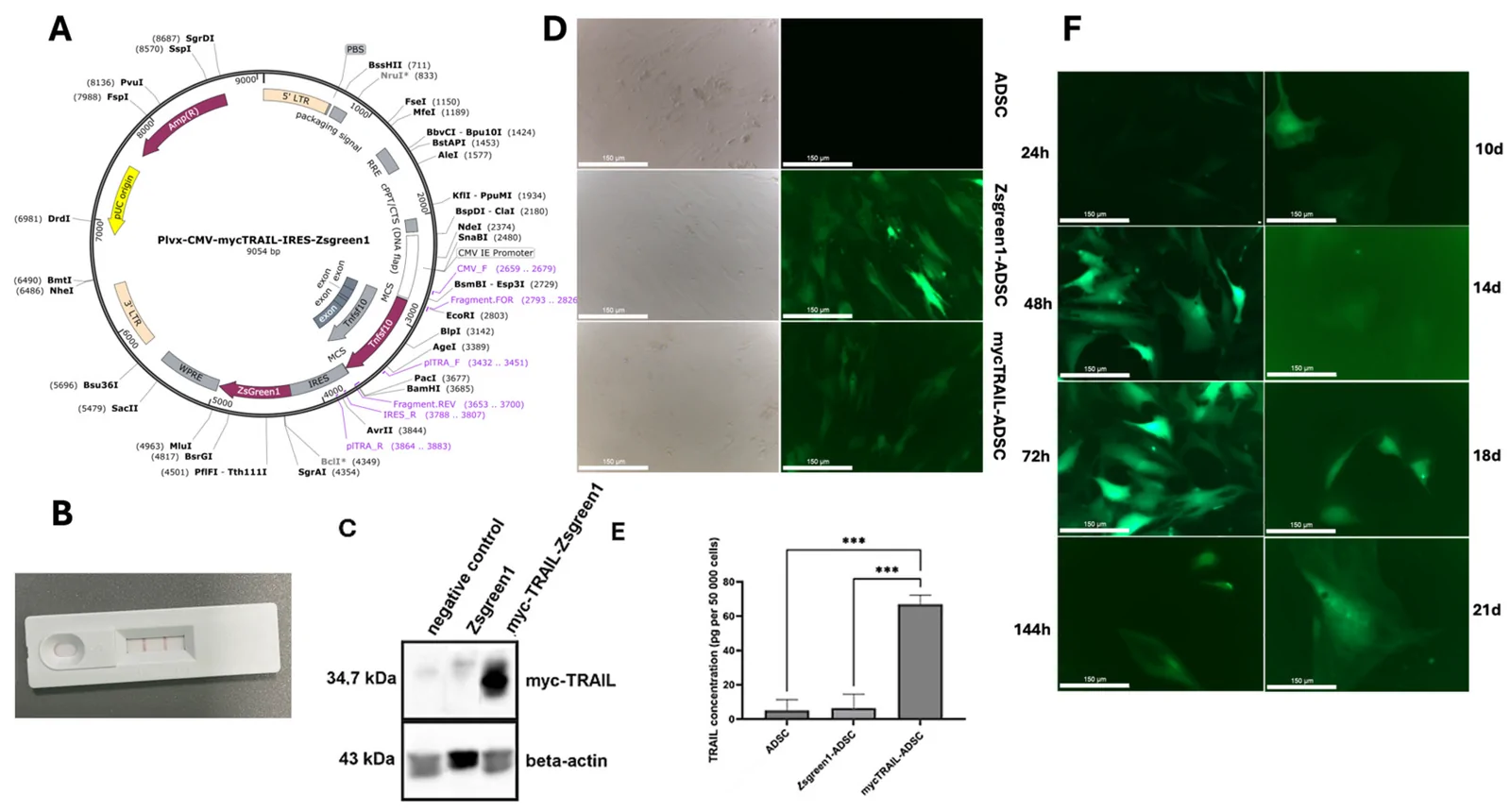
(**A**) Plasmid containing rat TRAIL gene (NM_145681.2) with N-terminal myc tag was cloned into Plvx-CMV-IRES-Zsgreen1 vector. Created using SnapGene^®^ software 7.1.2 (from Dotmatics; available at snapgene.com). (**B**) p24-based ELISA (Lenti-X Go-Stix) detecting the lentiviral capsid protein p24 was used to verify the production of lentiviral particles. Here, ~5 × 10^6^ IFU/mL (**C**) myc-TRAIL protein was present in transfected HEK293T cells used for lentivirus production (50 μg per lane). Detected using anti-myc antibody. Note: lane loading variation reflects differential transfection efficiency across conditions rather than inconsistent protein isolation; beta-actin loading control bands are shown in Figure S3 confirming comparable total protein input. (**D**) rADSCs were transduced using lentiviral supernatant (MOI = ~100) produced using VSV Single Shots in HEK293T cells using previously made plasmid. Pictures taken at t = 72 h of Plvx-mycTRAIL-IRES-Zsgreen1 transduced, Plvx-Zsgreen1 transduced and negative control cells. Scale bar is 150 µm. (**E**) rADSCs were transduced (pooled sample, MOI ≈ 30, 100, 160). Cells at 72 h post-transduction (three pooled wells of a 24-well plate; total 150,000 cells) were collected, and rat TRAIL concentration was determined relative to a standard curve. The experiment was performed as three independent biological replicates (separate transductions and passages), each with technical duplicates that were averaged before analysis; individual biological replicate values are overlaid. Data are mean ± standard deviation. One-way ANOVA was used to compare groups. *** *p* < 0.001. The read-out is total cell-associated TRAIL measured in freeze–thaw lysates, not soluble TRAIL. (**F**) Myc-TRAIL-Zsgreen1 rADSC were cultured for up to 21 days to assess long-term transgene expression. Reporter fluorescence was quantified as mean fluorescence intensity per field (ImageJ 1.53v) across multiple fields per time point and is plotted as a decay curve alongside representative micrographs; intensity declined markedly after approximately six days. Reporter fluorescence is an indirect proxy for TRAIL protein, as Zsgreen1 is expressed from the same IRES-containing transcript. Scale bar is 150 µm.

**Figure 3:**
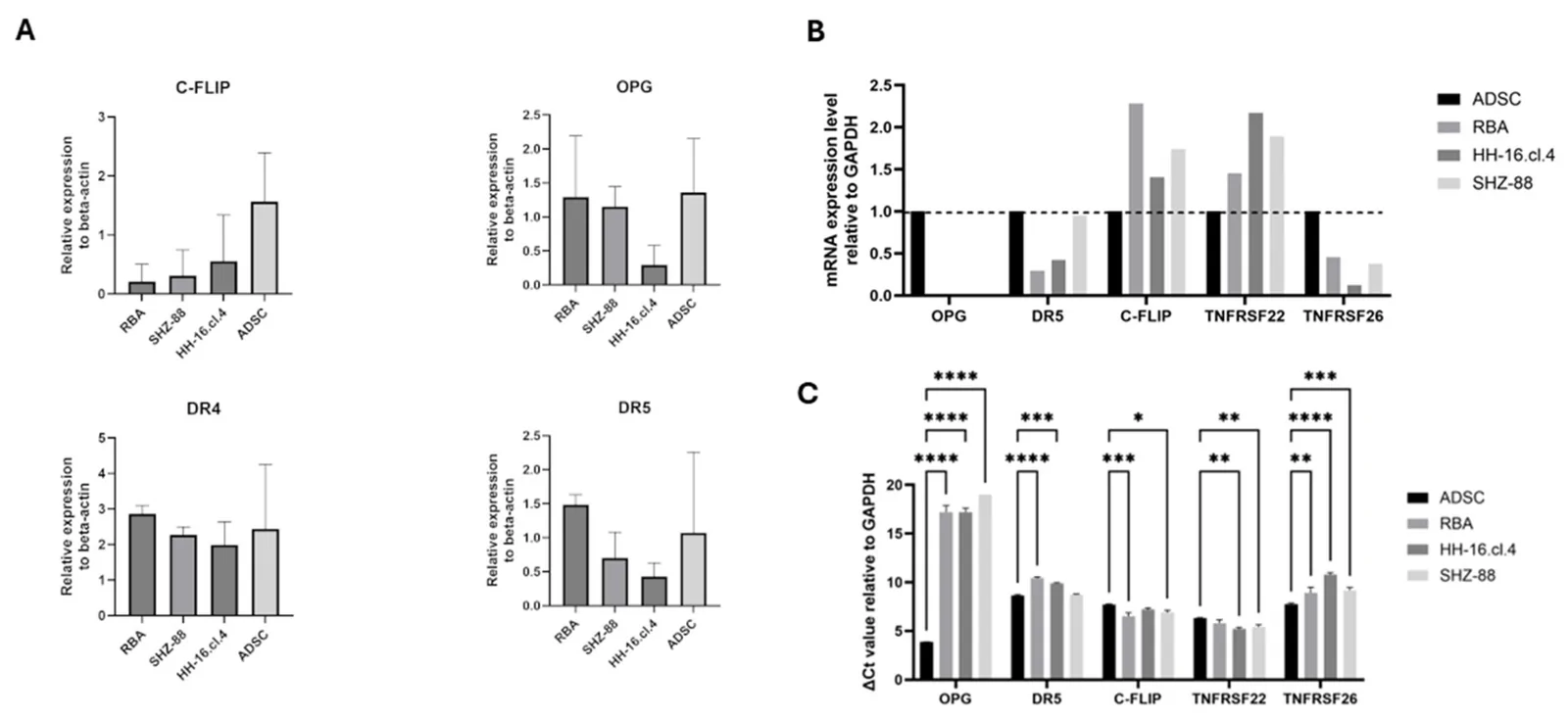
(**A**) The expression of TRAIL-signalling-pathway-associated proteins in rADSCs and different rat mammary gland cancer cell lines (SHZ-88, HH-16.cl.4, RBA) established using Western blot, repeated three times (bar graph shows densitometric quantification normalized to beta-actin; representative blot images are provided in Figure S3). (**B**) The expression of TRAIL-signalling-pathway-associated proteins in rADSCs and rat mammary gland cancer cells presented as relative expression to GAPDH mRNA estimated using the 2^−∆∆Ct^ method. (**C**) The expression of TRAIL-signalling-pathway-associated proteins in rADSCs and rat mammary gland cancer cells presented as ΔCt value relative to GAPDH mRNA with standard deviation. The experiment was performed as three independent biological replicates, each in technical duplicates averaged within replicate before analysis; individual biological replicate values are overlaid. Two-way ANOVA with Dunnett’s multiple-comparisons test, *—*p* < 0.05, **—*p* < 0.01, ***—*p* < 0.001, ****—*p* < 0.00001.

A plasmid containing rat TRAIL gene with N-terminal myc tag (Figure 2A) along with Zsgreen1 green fluorescent protein was successfully generated using the cloning technique described in the Methods section and the correct sequence was confirmed using Sanger sequencing (Figure S1). Lentiviral particles were successfully generated and an estimated titre of ~5 × 10^6^ IFU/mL verified using p24 ELISA (Lenti-X Go-Stix) was generated using the described method (Figure 2B). The transgene was highly expressed in HEK293T cells used for lentivirus production (Figure 2C). No significant HEK293T cell death was observed during the 48 h lentiviral production period despite TRAIL overexpression, consistent with the known insensitivity of HEK293T cells to TRAIL-mediated apoptosis owing to their high endogenous expression of anti-apoptotic proteins. rADSCs were transduced using control fluorescent lentiviral supernatant (containing only fluorescent protein) or myc-tagged rat TRAIL lentiviral supernatant and the cells achieved the highest fluorescence intensity at 72 h post-transduction in both control transduced and myc-TRAIL transduced cells. Transduced rADSCs retained the spindle-shaped, plastic-adherent morphology of the parental population, continued to proliferate and remained viable throughout the culture period, and showed no self-directed cytotoxicity despite TRAIL production, consistent with the high osteoprotegerin and C-FLIP expression detected in these cells (Figure 3). Surface-marker immunophenotyping before and after transduction was not performed, so formal confirmation that the MSC immunophenotype is preserved after lentiviral modification remains outstanding (Figure 2D). Furthermore, at 72 h post-transduction, the cells were collected and the total cell-associated TRAIL concentration (intracellular plus membrane-anchored, measured in freeze–thaw lysates) was estimated at 66.2 pg per 50,000 cells, compared with 5.73 pg and 4.43 pg per 50,000 cells in control-transduced and non-transduced cells, respectively (one-way ANOVA, *p* < 0.05) (Figure 2E). Finally, to verify long-term expression of the transgene, cells were cultured for up to 21 days and reporter fluorescence was quantified by measuring mean fluorescence intensity per field in ImageJ across multiple fields per time point. Fluorescence intensity declined markedly after approximately six days. Because Zsgreen1 is expressed from the same IRES-containing transcript as myc-TRAIL, reporter intensity is a reasonable proxy for transgene expression, but it reports transcriptional output rather than the quantity of functional TRAIL proteins (Figure 2F).

### 3.2. The Expression of TRAIL-Pathway-Associated Proteins Is Different in rADSCs Compared to Rat Mammary Gland Cancer Cells

Although ADSCs have been previously used as a biological vector to deliver human TRAIL into the tumour microenvironment, the differences in TRAIL-mediated apoptosis inhibitors (OPG, C-FLIP, TNFRSF22, TNFRSF26) and receptors (DR4, DR5) between ADSCs and mammary gland cancer cells have not been studied, especially in rat cells. As we quantified both expression at the RNA level and protein, we were able to more accurately estimate the expression differences between rat ADSCs and mammary gland cancer cells. rADSCs had a significantly higher expression of OPG, DR4 and DR5 compared to mammary gland cancer cell lines, confirmed at both mRNA and protein levels (Figure 3A,B and Figure S3). On the other hand, the expression pattern was similar in all mammary gland cancer cell lines in all molecules apart from DR5, whose mRNA level was almost as high as in SHZ-88 cells. All mammary gland cancer cell lines had higher mRNA levels of TNFRSF22 and lower of TNFRSF26 compared to rADSCs; however, these differences could not be studied on the protein level. Noteworthy, we noticed discrepancies between protein expression and RNA abundance in case of OPG in two out of three (RBA and SHZ-88) mammary gland cancer cell lines in which OPG was abundant at the protein level, yet RNA levels were similar to those of HH-16.cl.4 and RBA cells. Interestingly, C-FLIP expression was also pronounced in rADSCs on the protein level, yet less evident in mRNA analysis.

### 3.3. Two of Three Mammary Gland Cancer Cell Lines Are Susceptible to Recombinant Rat TRAIL

**Figure 4:**
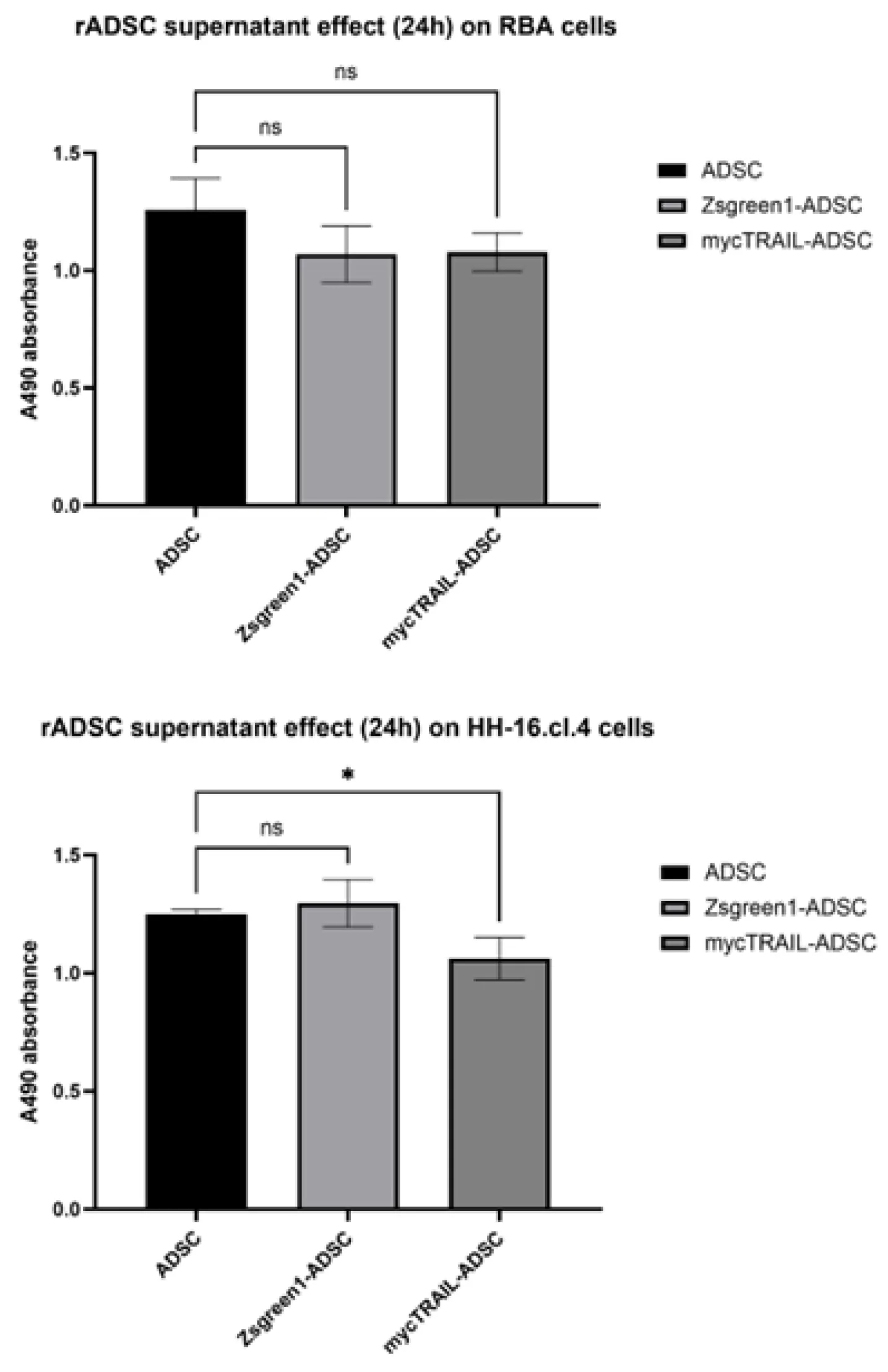
Proliferation of TRAIL-susceptible mammary gland cancer cells (RBA and HH-16.cl.4) after 24 h exposure to supernatant from transduced rADSCs collected 72 h post-transduction. The assay was performed as three independent biological replicates (separate transductions and passages), each with technical quadruplicates that were averaged within replicate before analysis; individual biological replicate values are overlaid. Data are mean ± standard deviation. One-way ANOVA; ns, *p* ≥ 0.05. Because the construct encodes membrane-anchored TRAIL, this cell-free conditioned-medium assay also serves as an indirect measure of the soluble TRAIL fraction. *—*p* < 0.05.

We further tested the susceptibility of rat mammary gland cancer cells to recombinant rat TRAIL at concentrations ranging from 0 to 100 ng/mL for 24 h (Figure S4). This assay was conducted as a qualitative screen to identify which cell lines respond to rat TRAIL, and not as a formal dose–response study; accordingly, no IC50 was calculated. Two of the three lines (RBA and HH-16.cl.4) showed reduced viability, whereas SHZ-88 was largely unaffected. Because this screen was performed as a single experiment in technical triplicate and the response across concentrations was non-monotonic, the absolute viability values are interpreted only qualitatively. An exploratory screen of RBA cells across a wider concentration range gave a similarly non-monotonic pattern. To avoid over-interpretation, this exploratory screen is presented in the Supplementary Materials rather than as a main figure. On the basis of this screen, we selected the two TRAIL-susceptible lines (RBA and HH-16.cl.4) for the subsequent experiments with myc-TRAIL-transduced rADSCs. We first collected supernatants from transduced rADSCs at 72 h post-transduction and applied them to the cancer cells for 24 h (Figure 4). The supernatant from myc-TRAIL-transduced rADSCs produced only a small reduction in viability that did not reach statistical significance in either mammary gland cancer cell line. Because the construct encodes full-length, membrane-anchored TRAIL, soluble factors in cell-free supernatant would be expected to deliver little active ligand; this result therefore motivated the subsequent contact-based co-culture experiments, in which transduced rADSCs and cancer cells were placed in direct or close proximity.

### 3.4. mycTRAIL-Transduced rADSCs Increase Cancer Cell Death in Both Direct and Indirect Co-Culture

**Figure 5:**
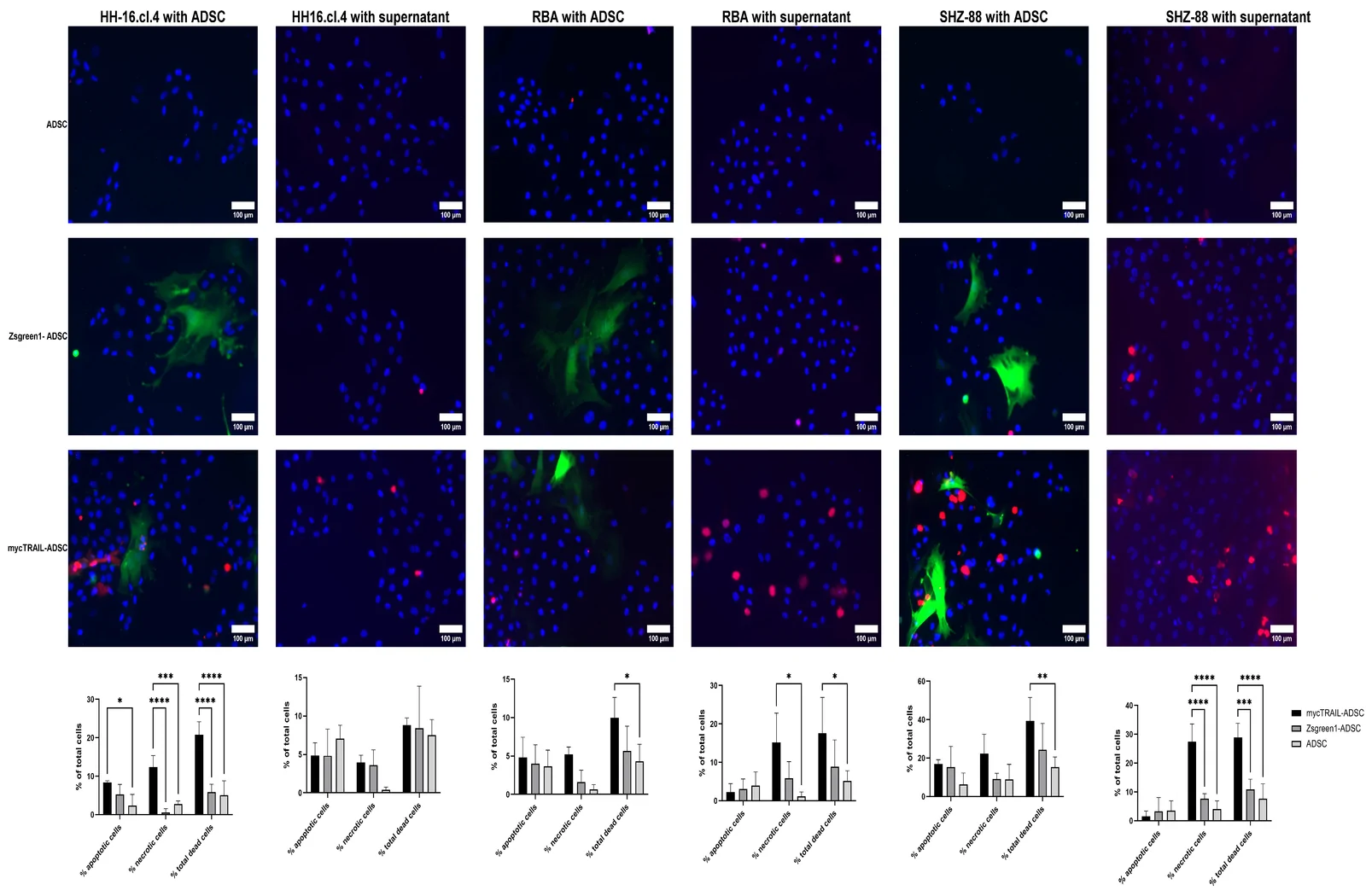
mycTRAIL-transduced ADSCs increase the death of mammary gland cancer cells in both direct and indirect co-culture. Fluorescent microscopy results of 24 h co-culture of mammary gland cancer cells with ADSC or with 72 h supernatant of ADSCs. Green cells represent Zsgreen1 or mycTRAIL-transduced ADSCs, while red cells represent necrotic cells and strongly blue cells represent apoptotic cells. Two-way ANOVA with Tukey’s multiple-comparisons test. *—*p* ≤ 0.05, **—*p* ≤ 0.01, ***—*p* ≤ 0.001, ****—*p* ≤ 0.0001. Cells with condensed, strongly stained blue nuclei were scored as apoptotic and propidium-iodide-positive cells as necrotic; these are morphological classifications and do not by themselves establish the mechanism of death. Scale bar is 100 µm.

To determine whether the growth-limiting effect of mycTRAIL-rADSC was accompanied by increased cancer cell death, we performed both direct and indirect co-culture experiments and scored live, apoptotic, and necrotic cells by Hoechst 33342/propidium iodide staining. Cancer cells co-cultured with mycTRAIL-ADSC showed a higher proportion of apoptotic, necrotic, and total dead cells than cancer cells treated with Zsgreen1-ADSCs or untransduced ADSCs (two-way ANOVA, *p* ≤ 0.05; Figure 5). A similar pattern, with smaller differences, was observed for supernatant-treated cells. The magnitude of the effect varied between cell lines, with the largest increase in apoptotic and necrotic fractions seen in HH-16.cl.4. The concurrent presence of both propidium-iodide-positive (necrotic) and condensed-nuclei (apoptotic) cells indicates a mixed mode of cell death rather than apoptosis alone. We additionally probed cleaved caspase-3 by Western blotting at the 24 h time-point but did not detect consistent differences between the treatment groups (Supplementary Figure S5). This is most plausibly explained by the transient nature of caspase-3 cleavage, which a single 24 h lysate may miss, and by the necrotic component evident in the imaging data; we therefore base the cell-death conclusion on the morphological (Hoechst/PI) read-out rather than on caspase-3.

### 3.5. Long-Term Effects of mycTRAIL-Transduced rADSCs on the Proliferation of TRAIL-Susceptible Mammary Gland Cancer Cells

**Figure 6:**
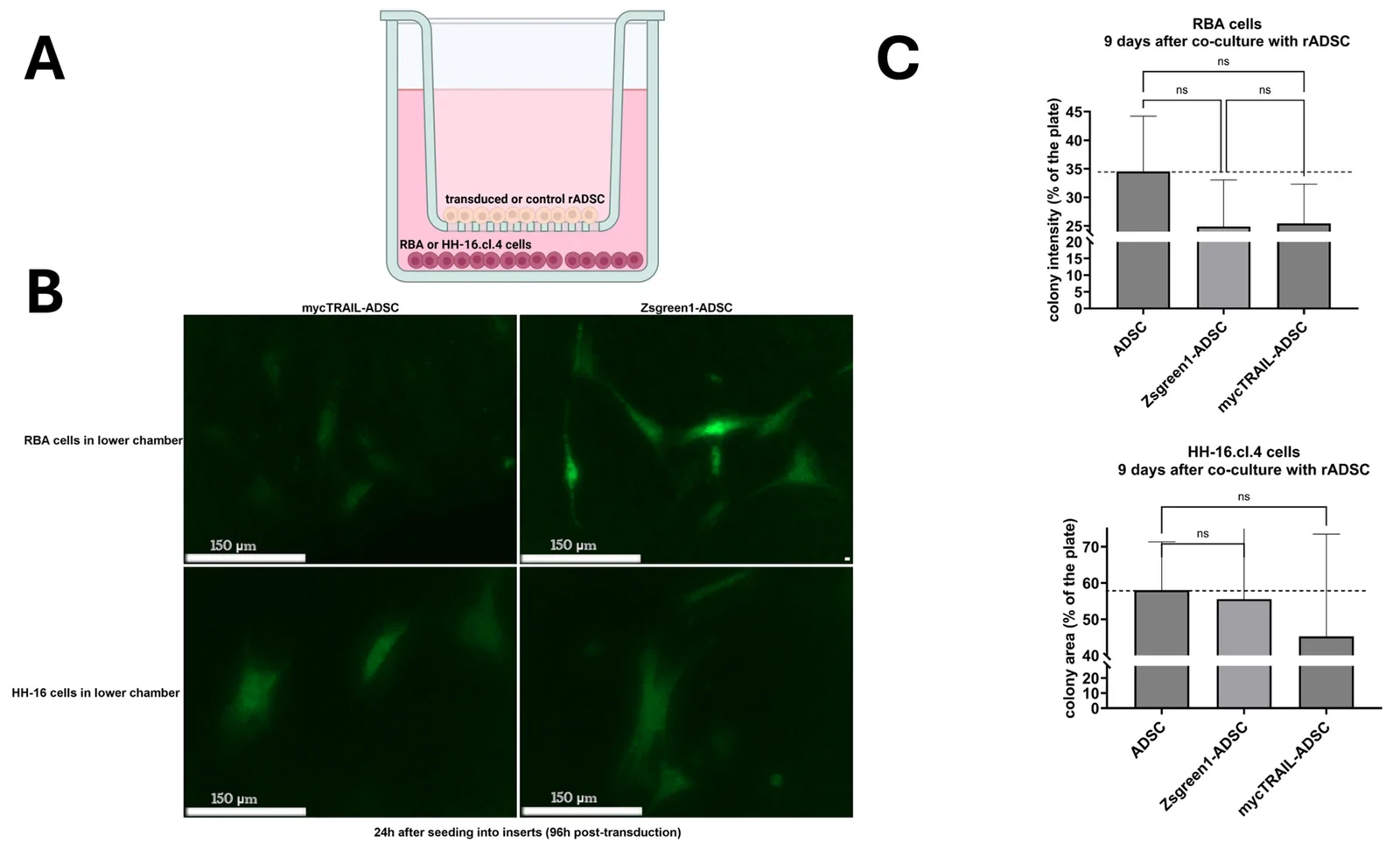
(**A**) A diagram presenting the method used for co-culture studies. Created in BioRender. Pascal, W. (2026) https://BioRender.com/e15o012. (**B**) Indirect co-culture of transduced rADSCs and mammary gland cancer cells (RBA or HH-16.cl.4) using 3 um co-culture inserts. Representative fluorescent pictures of upper chamber 24 h after seeding transduced rADSCs. Scale bar is 150 µm. (**C**) Mammary gland cancer cells (RBA and HH-16.cl.4) were co-cultured with rADSCs together for 9 days and afterwards cells in lower chamber were stained using crystal violet. The experiment was performed as three independent biological replicates (separate transductions and passages), each with technical duplicates averaged within replicate before analysis; individual biological replicate values are overlaid and data are presented with standard deviation. The pre-specified TRAIL-specific contrast is mycTRAIL-rADSC versus Zsgreen1-rADSC.

To assess the impact of rADSCs on rat mammary gland cancer cells, we conducted a co-culture experiment over 9 days (Figure 6A). We seeded the mammary gland cancer cells into the lower compartment and 24 h later seeded TRAIL-transduced or control rADSCs into the higher compartment (Figure 6B). At nine days, the highest cell counts were found in wells without rADSC inserts or with untransduced rADSCs, depending on the cell line, while wells treated with mycTRAIL-transduced rADSCs contained the lowest count for RBA and the second lowest for HH-16.cl.4. Importantly, the two cell lines behaved differently. For HH-16.cl.4, mycTRAIL-rADSC reduced cell numbers more than empty-vector (Zsgreen1) rADSCs, indicating a TRAIL-attributable effect. For RBA, however, empty-vector rADSCs reduced cell numbers to a degree comparable with mycTRAIL-rADSC, so the growth reduction in this line cannot be attributed to TRAIL and instead reflects an effect of the transduced ADSCs themselves (Figure 6C).

## 4. Discussion

Our results show that rADSCs derived from inguinal fat pad retain their morphology after lentiviral transduction. This source of ADSCs was chosen for its accessibility and its well-established use in rat models [23]. Furthermore, this source of ADSCs most closely corresponds to potential clinical use, as subcutaneos fat deposits are the easiest source of ADSCs for isolation and expansion in an everyday setting [24]. Our results show that such transduction allows for long-term overexpression (up to 21 days, yet with significant efficiency reduction after 6 days) of rat TRAIL in rat ADSCs. Similar results have been achieved in the past using other lentiviral-based technologies [25], but thanks to the transduction with a fluorescent protein alongside transgene, the expression pattern overtime could have been observed.

Furthermore, we were able to describe the differences in the expression pattern of TRAIL-signalling-pathway-associated molecules in ADSCs and mammary gland cancer cells. Our results are not mechanistic, as we only studied the expression of a group of proteins in a basal state, yet among other changes we noticed a major difference between osteoprotegerin expression between rADSCs and cancer cells. This may explain the resistance of both rADSCs and one of the cancer cell lines (SHZ-88) to TRAIL may be mediated at least partially by the overexpression of OPG. Such results support the data gathered by Brualla et al. [26], who showed that ADSCs are resistant to TRAIL due to unknown reasons. Furthermore, in case of osteoprotegerin, we noticed a difference between protein abundance and RNA level in TRAIL-resistant mammary gland cancer cells (SHZ-88), which requires further examination and may occur due to post-transcriptional mechanisms such as increased mRNA stability or enhanced translation efficiency. Furthermore, this corresponds to the fact that among three cell lines used, SHZ-88, although untested for the presence of progesterone and estrogen receptors, most closely corresponds to the PR+/ER+ models due to its slower growth and cluster-forming behaviour, while two others: RBA and HH-16.cl.4, have features similar to triple-negative BC and HER2+ BC, respectively. This may explain such differences and is supported by a histological sample study [27], where PR/ER+ expression was positively correlated with OPG expression. Because hormone-receptor and HER2 status were not determined experimentally in these rat lines, the subtype correspondence proposed here is inferential and should be regarded as a hypothesis generated by, rather than a conclusion of, the present study. In case of other molecules, although death receptors (DR4 and DR5) were expressed at a lower level in cancer cells compared to ADSCs, TNFRSF22—assumed to be a decoy receptor [28]—was also expressed at higher levels in cancer cells, and it was not enough to induce resistance to TRAIL. Interestingly, TNFRSF26, an orphan receptor with an unknown endogenous ligand based on Conserved Domain Database data [29] has been found to be highly expressed in rADSCs compared to cancer cells, which could also contribute to the TRAIL-susceptibility status; therefore, determining its function is necessary.

We established the susceptibility of three rat mammary gland cancer cell lines for susceptibility to exogenous rat TRAIL in a qualitative screen, identifying two susceptible lines (RBA and HH-16.cl.4) that were carried forward to test rTRAIL-rADSC. We used two complementary approaches to probe direct cytotoxicity. First, supernatants from transduced cells produced only a small, non-significant reduction in the proliferation of mammary gland cancer cells. We then assessed the ability of rTRAIL-rADSC to limit mammary gland cancer cell growth in insert co-culture. The co-culture assays produced stronger inhibition than the supernatant assay, consistent with the use of full-length, membrane-anchored TRAIL, which acts most effectively through cell-to-cell contact rather than as a soluble factor. This suggests that, for future applications, transduced cells should be delivered in close proximity to the target tumour. Despite reports from other studies [30,31], we did not observe increased tumour cell growth attributable to rADSC administration. A notable caveat concerns the specificity of the effect. The co-culture experiments included a full control series—cancer cells alone, untransduced rADSCs, Zsgreen1-rADSCs (empty-vector cells carrying an identical transduction but no TRAIL) and mycTRAIL-rADSC—so that the difference between mycTRAIL-rADSC and Zsgreen1-rADSC isolates the TRAIL-attributable effect, and the difference between Zsgreen1-rADSC and untransduced rADSCs isolates the transduction-attributable effect. Interpreted through these pre-specified contrasts, the effect proved cell-line-dependent. This limitation is most evident in the RBA line, where empty-vector (Zsgreen1) rADSCs reduced cell numbers to an extent comparable with mycTRAIL-rADSC; the growth reduction in RBA therefore cannot be ascribed to TRAIL. A TRAIL-attributable effect was clearer in HH-16.cl.4, where mycTRAIL-rADSC outperformed the empty-vector control. The vehicle effect in RBA may reflect transduction-induced changes in ADSC signalling and secretory behaviour, as reported in numerous studies. Taken together, these data support a TRAIL-attributable effect in HH-16.cl.4 but not in RBA, where cells carrying the identical transduction without TRAIL produced a comparable reduction. The three cell lines also provide an internal specificity check: SHZ-88, identified in our screen as TRAIL-resistant and expressing the highest osteoprotegerin with low DR4/DR5, would be expected to respond to a non-specific effect of rADSCs or to the transduction procedure but not to TRAIL itself. The magnitude of any TRAIL-specific benefit in this homologous rat system is therefore modest and cell-line-dependent, and definitive attribution would require ligand-neutralization or death-receptor-blockade experiments [32]. Regarding the mechanism, our data show increased cancer cell death in both direct co-culture and with the supernatant, with morphological features compatible with both apoptosis and necrosis. Because cleaved caspase-3 did not differ consistently between groups, we cannot conclude that apoptosis is the principal mechanism: the concurrent presence of propidium-iodide-positive cells indicates a mixed mode of death, and the single 24 h sampling point may in addition have fallen outside the transient window of caspase-3 activation. Definitive mechanistic attribution would require a time-resolved quantitative apoptosis assay, such as Annexin V/propidium iodide flow cytometry with caspase inhibition.

This pilot study has several limitations. First, the therapeutic effect was measured mainly with non-specific colorimetric assays, which may partly explain the lower-than-expected efficacy of rTRAIL-rADSC. Second, several findings are of limited conclusiveness because the small effect sizes did not reach statistical significance, and they therefore require confirmation in larger studies. Third, transgene expression declined markedly after approximately six days. This is a central constraint of the strategy rather than an incidental observation: it implies that a single administration of transduced cells delivers a therapeutic window of days rather than weeks, and that any in vivo application would require antibiotic selection before delivery (not feasible in the present setup), repeated dosing, or a vector giving more durable expression. Because reporter fluorescence is an indirect measure of TRAIL protein, the true functional half-life of TRAIL production may be shorter still. Fourth, although the design included both untransduced and empty-vector (Zsgreen1) rADSC controls, the TRAIL-specific contrast reached a clear separation only in HH-16.cl.4, so that in RBA the contribution of the ADSCs and of the transduction procedure could not be distinguished from that of TRAIL. Fifth, several experiments were repeated a limited number of times and inference is correspondingly cautious; the immunophenotype of the transduced vehicle cells was not formally verified; and soluble and cell-surface TRAIL were not quantified directly, so the functionally available fraction of the ligand remains incompletely defined. Finally, this study was restricted to in vitro models. The insert co-culture system approximates only the spatial relationship between the therapeutic cells and the tumour, and does not reproduce the cellular heterogeneity, extracellular matrix, vasculature or immune compartment of the tumour microenvironment; in vivo studies are therefore required to establish the therapeutic potential of rat TRAIL-transduced rADSCs.

All in all, biologic therapy based on rat TRAIL overexpression delivered via lentivirally transduced rADSCs is feasible, yet only moderately efficient against susceptible rat mammary gland cancer cell lines. Furthermore, this limited efficacy points towards a question: whether rat TRAIL is as effective in inducing apoptosis and limiting cancer cell growth as human TRAIL. As this is the first study aimed at creating a fully translational model (rat TRAIL-transduced rat ADSCs) for in vivo studies, it shows the limitations of such an approach due to unexpectedly moderate effects.

We believe that developing further therapies based on biologic brachytherapy, i.e., using molecular biology tools to enhance production and expression of certain biological agents, in limited tissue bulks, is the next step in case of transduced rADSCs’ effects against breast cancer, and potentially with other anticancer agents. Further studies on the potential of rADSCs for cancer treatment may bring new therapies to patients in the future.

## 5. Conclusions

This is the first study to report a fully homologous, in vitro TRAIL-based therapy in which rat-adipose-derived stem cells deliver rat TRAIL to rat mammary gland cancer cells. In this pilot study, the therapeutic effect was moderate and depended on the TRAIL susceptibility of the cancer cell line; it was separable from the effect of the transduced vehicle in HH-16.cl.4 but not in RBA. This limited efficacy raises the question of whether rat TRAIL is a suitable homologue of human TRAIL in animal models. Our data suggest that the effect is spatially confined to the immediate vicinity of the transduced cells. Nonetheless, as a prolonged and precise local adjunct therapy (biologic brachytherapy), the approach shows promise. Animal studies are now needed to confirm its efficacy.

## Data Availability

The raw data are available from corresponding author upon request.

## Supplementary Materials

The following supporting information can be downloaded at: https://www.mdpi.com/article/10.3390/cimb48090941/s1.
