# Supplementary material for: Rat TNF-Related Apoptosis-Inducing Ligand (rTRAIL)-Expressing Rat-Adipose-Derived Stem Cells (rADSC) Moderately Limit Growth of Rat Mammary Cancer Cells—A Pilot Study
Source: Curr Issues Mol Biol. 2026 Sep 15;48(9):941. doi: 10.3390/cimb48090941 (PMC13605116; doi:10.3390/cimb48090941)

Figure S1 - plasmid Plvx-CMV-myc-TRAIL-Zsgreen1 sequence alignment confirmation

Both MG001 and MG002 are perfectly aligned plasmid Plvx-CMV-myc-TRAIL-IRES-Zsgreen1. Can be used for transfection/transduction.

<u>MG002</u> – perfect sequence both using CMV_F and IRES_R – no mismatches/inserts

Beggining of myc-tagged TRAIL (CMV_F):

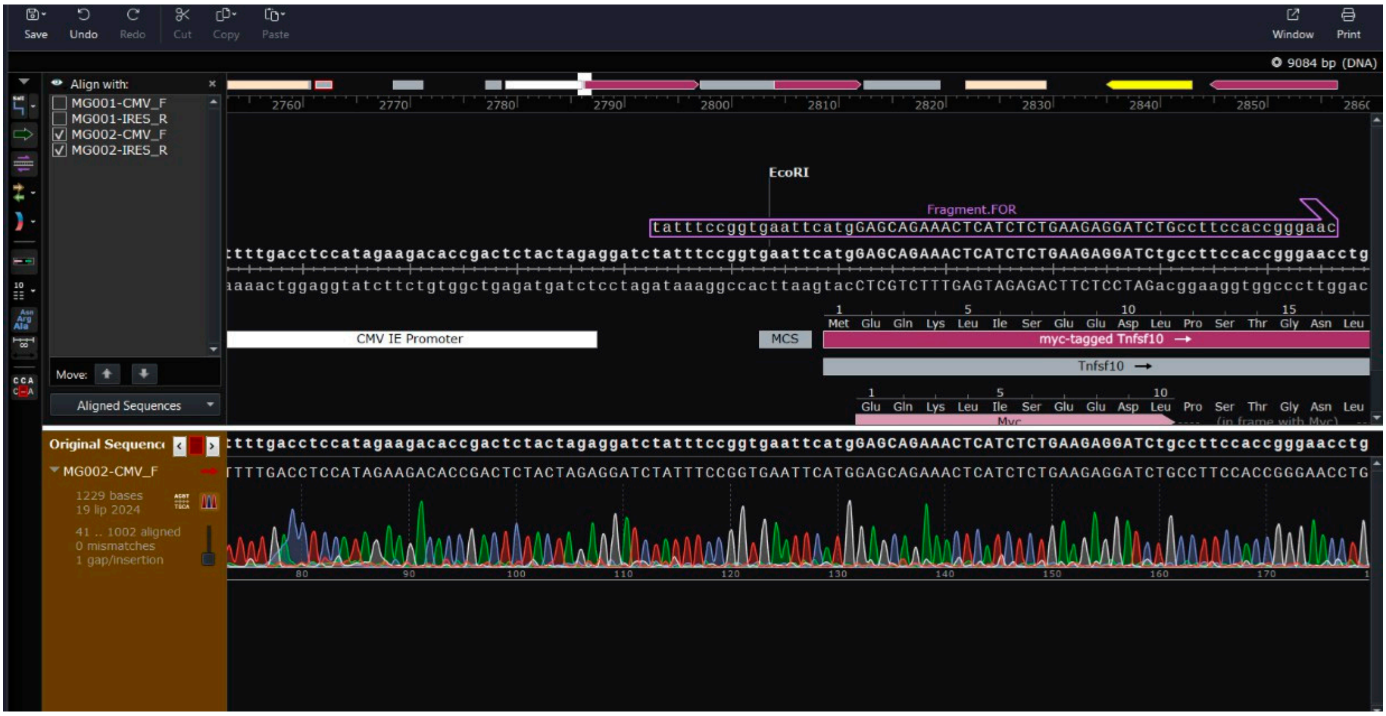

End of myc-tagged TRAIL (IRES_R and CMV_F):

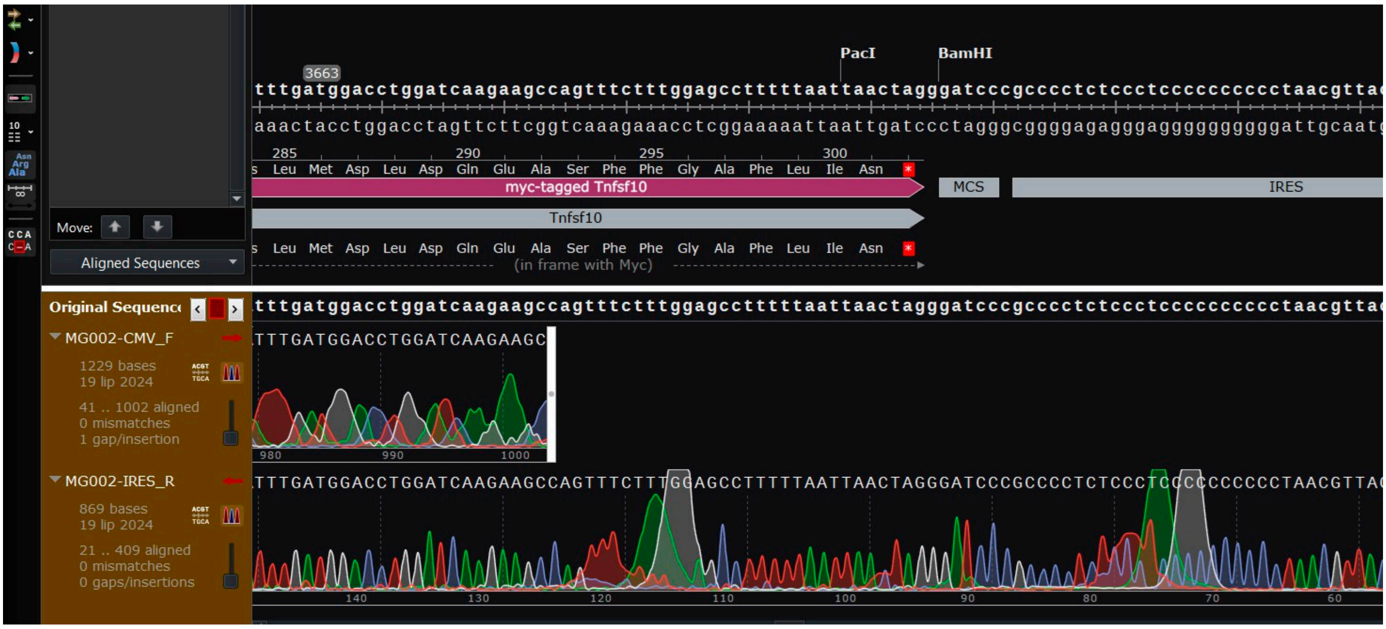

MG001 – two potential mismatches (CMV_F) but first one: not possible because it would affect EcoR1 digestion site which works – C into T

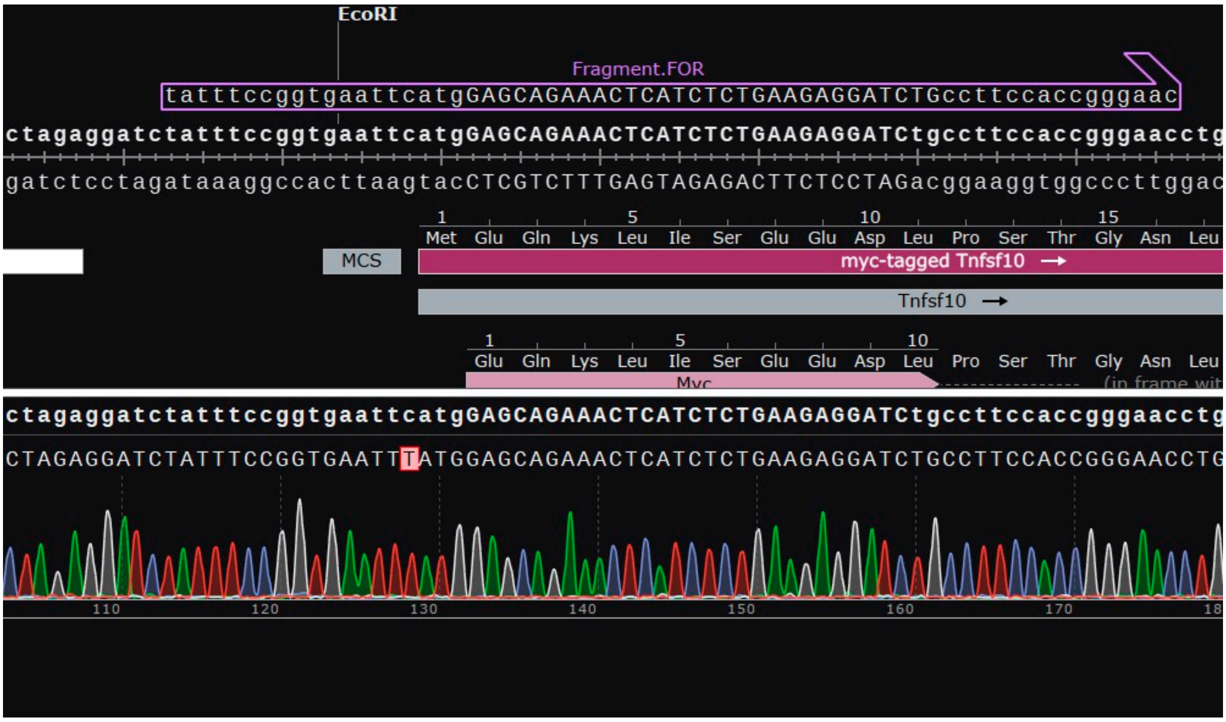

Second mismatch (G into C) → machine mistake

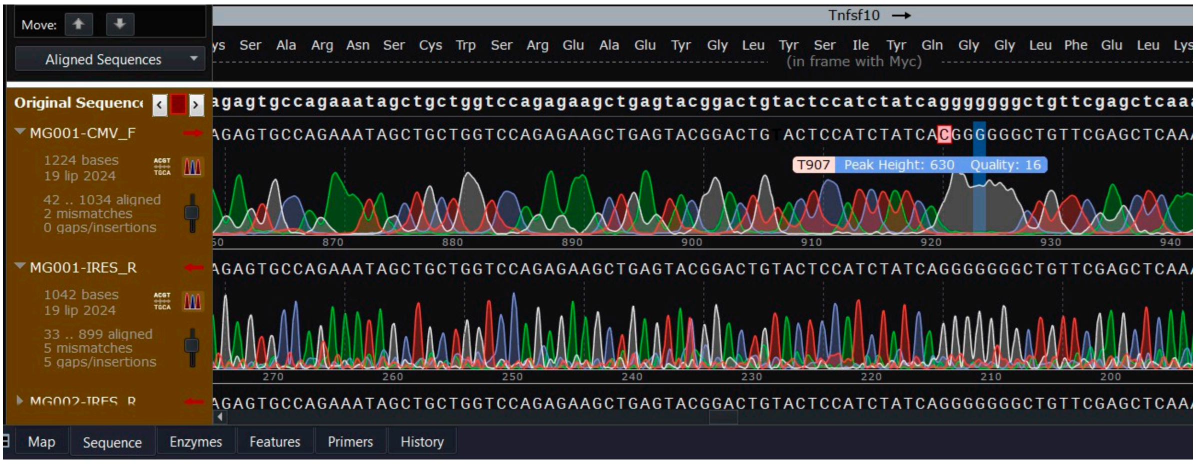

Figure S2: mycoplasma test result

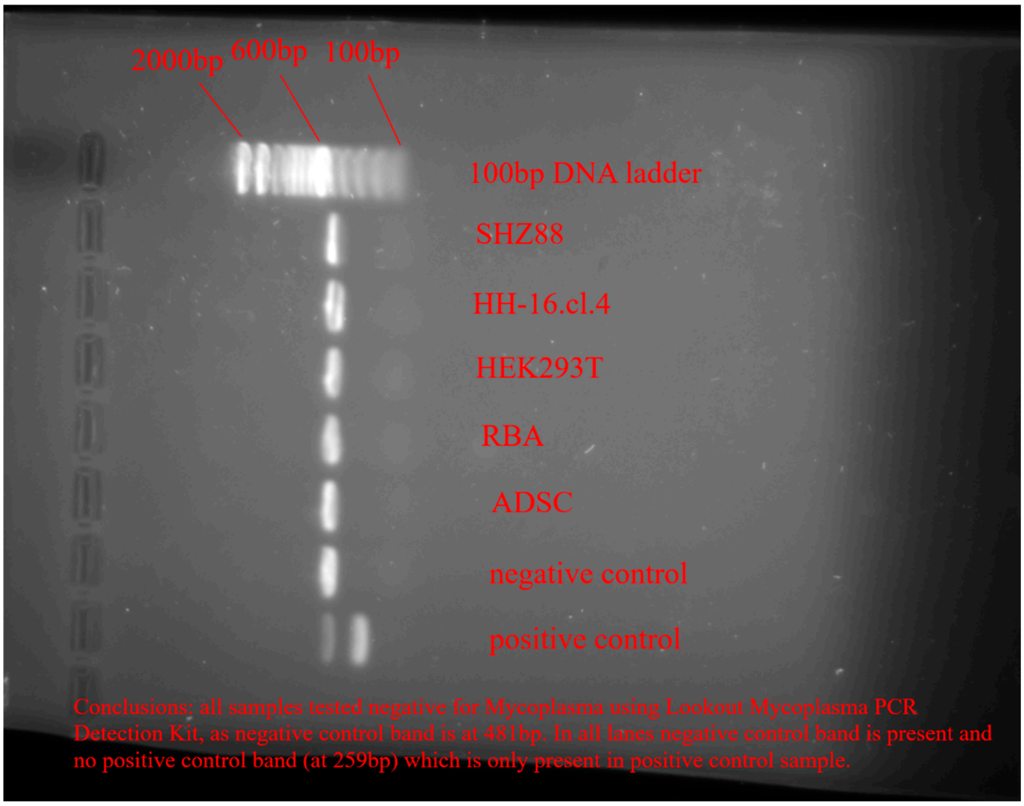

Figure S3 - Representative blot of protein analysis related to TRAIL signaling in examined cell lines

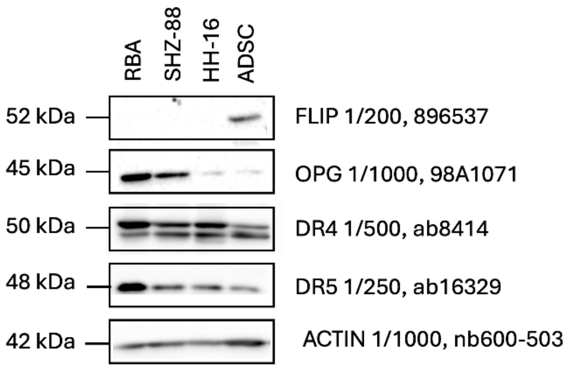

10ug cell lysate per lane

Secondary antibody for:
FLIP/OPG – anti-mouse 1/1000
DR4/DR5/ACTIN – anti-rabbit 1/1000

Figure S4 - Proliferation assay using exogenous rat TRAIL (0-100ng/ml) to which mammary gland cancer cells were exposed for 24h. Assay was done once in triplicates and the results are presented with standard deviation.

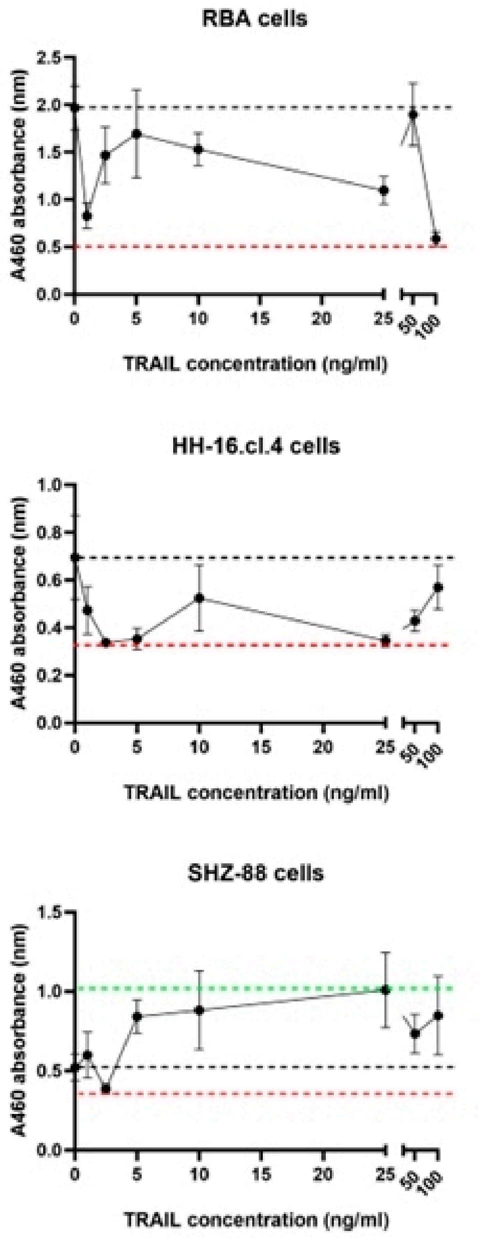

Figure S5 - Expression of cleaved caspase-3 relative to beta-actin in directly co-cultured mammary gland cancer cells and ADSC or in 72-hour supernatant treated mammary gland cancer cells

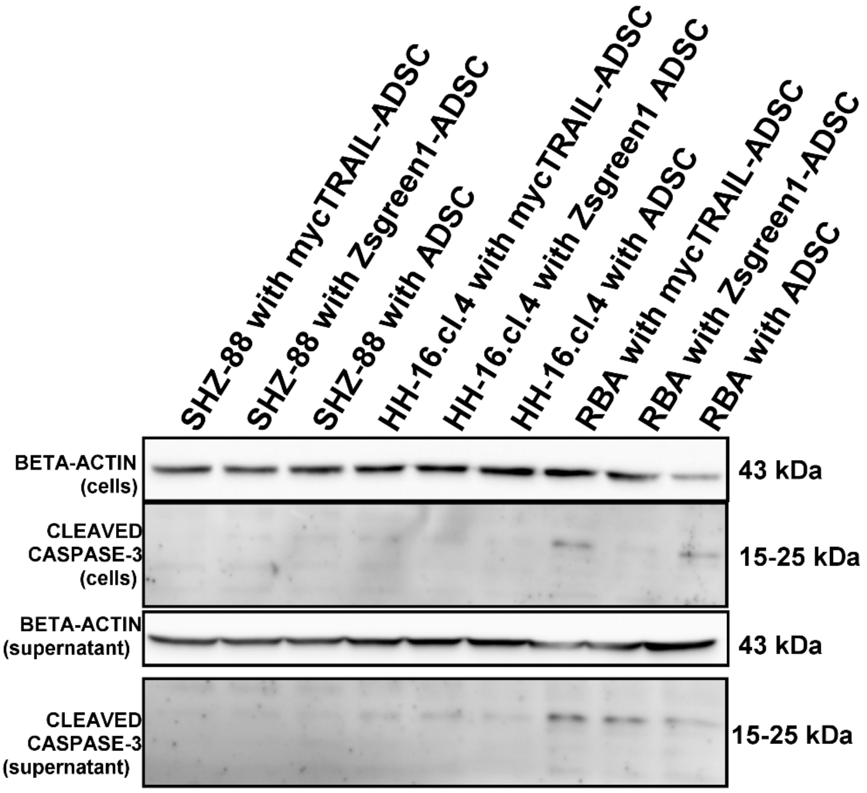

Supplement: Supplementary file 1 [file cimb-48-00941-s001.zip › cimb-4477557-supplementary.pdf]
